# Telbivudine on IgG-associated hypergammaglobulinemia and TGF-β1 hyperactivity in hepatitis B virus-related liver cirrhosis

**DOI:** 10.1371/journal.pone.0225482

**Published:** 2019-11-26

**Authors:** Cheng-Hsun Ho, Ting-Tsung Chang, Rong-Nan Chien

**Affiliations:** 1 Department of Medical Laboratory Science, College of Medicine, I-Shou University, Kaohsiung, Taiwan; 2 Department of Internal Medicine, National Cheng Kung University Hospital, College of Medicine, National Cheng Kung University, Tainan, Taiwan; 3 Institute of Molecular Medicine, College of Medicine, National Cheng Kung University, Tainan, Taiwan; 4 Department of Hepatogastroenterology, Chang Gung Memorial Hospital, Linkou, Taiwan; Kaohsiung Medical University Chung Ho Memorial Hospital, TAIWAN

## Abstract

As debate rumbles on about whether anti-hepatitis B virus (HBV) nucleos(t)ide analogue treatments modulate host immune system during end-stage liver diseases, we studied effects of two potent anti-HBV agents, telbivudine or entecavir, on humoral immune activities including cytokine secretion, immunoglobulin production, and IgG-Fc agalactosylation, which is known to induce proinflammatory responses, in liver cirrhosis. Serum IgG-Fc *N*-glycan structures in patients with HBV-related liver cirrhosis, who had received either telbivudine treatment or entecavir treatment for at least 48 weeks were analyzed using liquid chromatography tandem-mass spectrometry. Levels of cytokines and each immunoglobulin isotype were measured using enzyme-linked immunosorbent assays. Results showed that 48 weeks of entecavir treatment caused HBV DNA loss, alanine aminotransferase normalization, and an amelioration of hypergammaglobulinemia in cirrhotic patients; however, telbivudine treatment, though possessing similar efficacies on HBV suppression and an improvement in liver inflammation to entecavir treatment, did not mitigate IgG-related hypergammaglobulinemia. Levels of IgG and transforming growth factor (TGF)-β1 in sera of the cirrhotic patients before and during treatment were positively correlated. *In vitro* assays revealed that telbivudine treatment induced TGF-β1 expression in human macrophagic cells. Moreover, recombinant TGF-β1 treatment stimulated cell proliferation and IgG overproduction in human IgG-producing B cell lines. Finally, we found that telbivudine treatment enhanced the proportion of serum IgG-Fc agalactosylation in cirrhotic patients, which was associated with enhanced levels of TGF-β1 and IgG. In conclusion, telbivudine therapy was associated with TGF-β1 hyperactivity, IgG-related hypergammaglobulinemia, and IgG-Fc agalactosylation in HBV-related liver cirrhosis.

## Introduction

Liver cirrhosis, resulting from an advanced liver fibrosis, is an end-stage disease that causes more than one million deaths in global per year. Hepatitis B or C virus infection, alcoholic or non-alcoholic fatty liver, autoimmune liver diseases, and hereditary metabolic liver diseases have been recognized as primary causes of liver fibrosis or cirrhosis [1, 2]. The cirrhotic state impedes various liver functions including albumin manufacture, bilirubin metabolism, and clotting factors synthesis, thereby leading to spontaneous bleeding, ascites, or edema [3]. Moreover, portal hypertension and esophageal varices are frequently seen in cirrhotic patients owing to an impaired endothelium-dependent relaxation in the intrahepatic/sinusoidal microcirculation and an increased intra-hepatic vascular resistance [4]. The diagnosis of liver cirrhosis is based on physical findings, histological examinations, or evidence from imaging modalities. However, cirrhosis in the initial stage (compensated cirrhosis) is often asymptomatic and hard to be detected.

Current medications are not able to cure cirrhosis that has already occurred. Pharmacotherapy on the cirrhotic population mainly targets the illness that led to cirrhosis or related complications to prevent or delay the worsening of cirrhosis before the development of liver failure or cancer. For the management of hepatitis B virus (HBV)-related liver cirrhosis, a long-term administration of antiviral nucleos(t)ide analogue, such as entecavir and telbivudine, is the standardized recipe [5]. We previously reported that a median exposure to entecavir therapy of approximately 6 years reverses liver fibrosis/cirrhosis [6], suggesting that antiviral therapy, with evidence of complete suppression of HBV viral loads and continual normalization of ALT, improves liver histology with accompanying regression of fibrosis. However, little is known about the efficacies of antiviral therapies on host immune modulation and cirrhosis-related complications, for example, hypergammaglobulinemia. Therefore, we conducted a cohort study plus cell-based assays to assess effects of antiviral treatment on host humoral immune events, thus to better understand the pathophysiology of complex comorbidities beyond the advancement of liver disorders as well as the development of a multidisciplinary diagnostic strategy for HBV-related liver cirrhosis.

## Materials and methods

### Patients

This retrospective study was approved by the Institutional Review Boards of National Cheng Kung University Hospital (ER0990385) and of Keelung Chang Gung Memorial Hospital (102-0459B). Written informed consent was obtained from each participant. The study protocol conforms to the ethical guidelines of the 1975 Declaration of Helsinki. Patients with HBV-related liver cirrhosis (n = 68), who had HBV surface antigen (HBsAg) for more than 6 months and HBV DNA >2000 IU/mL, were enrolled from outpatient clinics of both hospitals. Liver cirrhosis was diagnosed according to liver biopsy or classic ultrasound findings combined with esophageal varices, gastric varices, or splenomegaly [7]. Classic ultrasound findings in liver cirrhosis include nodular contour and coarse echotexture of the liver. All the patients were treatment-naive and received telbivudine (n = 29) treatment or entecavir (n = 39) treatment for at least 48 weeks. The fibrosis score, calculated by FIB-4 index, were available in 11 patients in the entecavir group and 12 patients in the telbivudine group at baseline, respectively and were available in 4 patients in the entecavir group and 9 patients in the telbivudine group after 48 weeks of treatment. Subjects who tested positive for hepatitis C virus, human immunodeficiency virus, alcoholic or autoimmune-induced liver diseases, rheumatoid arthritis, juvenile onset chronic arthritis, systemic lupus erythematosus, or Crohn’s disease were excluded [7].

#### Enzyme-linked immunosorbent assay (ELISA)

Total human serum IgA, IgD, IgE, IgG, and IgM were detected using ELISA Quantitation Sets (Bethyl Laboratories, Montgomery, TX). These kits have no cross-reactions to bovine γ-globulins. The level of total γ-globulin is the sum of five immunoglobulin isotypes. Levels of interleukin (IL)-1β, IL-4, IL-6, IL-8, IL-10, IL-12p70, IL-17A, IL-22, IL-27, interferon-γ, transforming growth factor (TGF)-β1, tumor necrosis factor (TNF)-α in serum from patients were detected using Ready-Set-Go ELISA kits (eBioscience, San Diego, CA) [8].

#### Cell culture and treatment

A human hepatoma cell line HepG2 (Cat. NO. 60177), a human monocytic cell line U-937 (Cat. NO. 60435), IgM-producing human B cell lines Ramos (Cat. NO. 60252) and CA46 (Cat. NO. 60511), and IgG-producing human B cell lines ARH-77 (Cat. NO. 60385) and IM-9 (Cat. NO. 60115), were purchased from Bioresource Collection and Research Center (Hsinchu, Taiwan), which were originated from American Type Culture Collection, and used at passages 2 to 4. HepG2 cells were cultured in Dulbecco's Modified Eagle's medium (Caisson Labs, Logan, UT). B lineage and U-937 cells were cultured in Roswell Park Memorial Institute-1640 medium (Caisson Labs) supplemented with 10% of heat-inactivated fetal bovine serum (Thermo Fisher Scientific, Paisley, UK), 100 U/mL of penicillin and 100 μg/mL of streptomycin at 37°C in the presence of 5% CO_2_. U-937 cells were treated with 100 ng/mL of phorbol 12-myristate 13-acetate (Sigma-Aldrich, St. Louis, MO) for 72 hours to induce macrophage differentiation. Entecavir and telbivudine were purchased from Abmole BioScience (Houston, TX) and AdooQ BioScience (Irvine, CA), respectively. Recombinant human TGF-β1 was purchased from R&D Systems (Minneapolis, MN). The number and viability of cells were calculated using EVE automatic cell counter (NanoEnTek Inc, Seoul, Korea).

#### Analysis of IgG-Fc N-glycan structure

Detection of serum IgG-Fc glycosylation pattern using liquid chromatography–tandem mass spectrometry (LC-MS/MS) has been described previously [7, 8]. Briefly, IgG proteins in sera or in culture media of IM-9 cells were purified using Protein G 4 Fast Flow Sepharose beads (GE Healthcare, Piscataway, NJ) and resolved using 10% sodium dodecyl sulfate polyacrylamide gel electrophoresis. The protein spot located between 50 and 55 kDa was excised from polyacrylamide gels, digested with 20 ng/μL trypsin (Promega, Madison, WI) in 10 mM ammonium bicarbonate at 37°C overnight, acidified by 1% trifluoroacetic acid, and extracted by vigorous vortexing. The tryptic peptides were then applied to LC-MS/MS analysis. IgG subclass 1 is the analytic target in the serum samples because it is the major component of serum IgG pool and possesses common almost all antibody responses and cytophilic properties. Selected ion chromatograms of different glycoforms attached to the peptide backbone (EEQYNSTYR) were extracted from the raw data. The peak height or peak area obtained from the extracted ion chromatogram of a particular glycoform of tryptic peptides was divided by the sum of all forms in the same LC-MS chromatogram. The percentage of each glycoform was determined from an average of three LC-MS/MS runs. Ten glycoforms on IgG-Fc were analyzed and other very low-abundant Fc glycoforms, such as tri-antennary, tetra-antennary, and highly mannosyl, were preliminarily excluded. Investigators were blinded to any information and experimental results of the patients when analyzing IgG *N*-glycans.

#### Quantitative reverse transcription-polymerase chain reaction (qRT-PCR)

Total RNAs were purified using REzol C&T (Protech Technology, Taipei, Taiwan) and reverse transcribed using Superscript III First-Strand Synthesis System (Thermo Fisher Scientific). PCR was performed using Power SYBR Green PCR Master Mix and StepOne Real-Time PCR System (Thermo Fisher Scientific). The PCR program was set in an initial step at 95°C for 10 minutes with subsequently 40 cycles at 95°C for 15 seconds and 56°C for 1 minute. Primers for detecting messenger RNAs of human β-1, 4-galactosyltransferases (*B4GALT*s) and glyceraldehyde 3-phosphate dehydrogenase, have been described previously [9, 10]. The Ct value of each PCR run was determined using StepOne Software version 2.3.

#### Statistical analysis

SPSS 17.0 for Windows was used for all statistical analyses. Nominal variables were compared using Fisher's exact tests or Pearson Chi-square tests. Continuous variables were compared using Student’s *t* tests or Mann-Whitney *U* tests for two independent groups and paired *t* tests for two related groups. Pearson’s correlation coefficient (*r*) was used to evaluate the relationship between parameters. Multivariant logistic regression analyses were conducted to evaluate factors that were associated with the hyperactivity or post-treatment increment of IgG in patients with liver cirrhosis. Significance was defined as *P* < 0.05, and all *P*-values were two-tailed.

## Results

### Clinical data of patients with HBV-related liver cirrhosis

Entecavir and telbivudine groups had even distributions of gender, age, HBV e antigen status, and Child-Pugh score and had similar levels of alanine aminotransferase (ALT), aspartate aminotransferase (AST), albumin, total globulins, γ-globulins, total bilirubin, and HBV DNA at baseline (Table 1). IgG and IgM account for approximately 80.2% and 14.0% of γ-globulins, respectively, in cirrhotic patients. Both 48 weeks of entecavir and telbivudine treatments decreased levels of ALT, AST, and HBV DNA and increased the level of albumin. The level of AST at week 48 in the telbivudine group was a little higher than the upper limit of normal (ULN). A virological response (undetectable HBV DNA in serum) at week 48 was detected in 71.8% and 86.2 of patients with entecavir and telbivudine treatments, respectively.

**Table 1:** Clinical data of patients with HBV-related liver cirrhosis.

	Entecavir group (n = 39)			Telbivudine group (n = 29)				
Variable	Baseline	Week 48	*P*-value^1^	Baseline	Week 48	*P*-value^2^	*P*-value^3^	*P*-value^4^
Male, no. (%)	24 (61.5)			22 (75.9)			.325	
Age (years)	54.3 ± 9.2			56.7 ± 12.0			.340	
ALT (U/L)	86.6 ± 103.5	31.3 ± 13.8	.002	116.7 ± 135.2	40.4 ± 30.6	.005	.301	.101
AST (U/L)	71.6 ± 57.4	33.4 ± 9.6	< .001	89.5 ± 91.9	45.1 ± 27.2	.013	.329	.034
Platelet (10^3^/μL)*	145.9 ± 64.1	146.8 ± 41.6	0.335	121.0 ± 22.0	147.2 ± 92.0	0.714	0.970	0.599
Albumin(g/dL)	3.9 ± 0.5	4.1 ± 0.5	< .001	4.0 ± 0.6	4.1 ± 0.5	.487	.314	.680
Total globulin (g/dL)	3.8 ± 1.4	3.2 ± 0.9	.021	4.1 ± 2.8	4.8 ± 1.5	.273	.543	< .001
Albumin/Globulin ratio	1.1 ± 0.4	1.4 ± 0.4	.002	1.3 ± 0.6	0.9 ± 0.3	.014	.342	< .001
γ-globulin (g/dL)	2.2 ± 0.6	1.5 ± 0.5	< .001	1.9 ± 0.6	1.9 ± 0.6	.977	.104	.002
IgG (g/dL)	1.7 ± 0.5	1.4 ± 0.5	< .001	1.6 ± 0.6	1.8 ± 0.6	.188	.345	< .001
IgA (g/L)	1.3 ± 0.9	0.5 ± 1.1	< .001	0.9 ± 0.8	0.2 ± 0.2	< .001	.095	.116
IgM (g/L)	3.2 ± 2.0	0.9 ± 0.6	< .001	2.5 ± 2.5	0.8 ± 0.4	< .001	.161	.512
IgD (μg/L)	3.0 ± 2.9	2.8 ± 2.5	.153	2.8 ± 2.8	3.1 ± 3.0	.577	.757	.648
IgE (μg/L)	2.7 ± 1.2	2.3 ± 0.9	.002	2.7 ± 1.2	2.6 ± 1.4	.595	.988	.236
Total bilirubin (mg/dL)	1.4 ± 1.1	1.1 ± 0.4	.202	1.3 ± 0.7	1.2 ± 0.5	.144	.922	.527
HBV DNA (Log_10_ IU/mL)	5.6 ± 1.4	0.9 ± 1.6	< .001	5.9 ± 1.2	0.4 ± 1.3	< .001	.438	.146
Virological response		28 (71.8)			25 (86.2)			.262
HBeAg (—), no. (%)	33 (84.6)	36 (92.3)	.480	29 (100.0)	29 (100.0)	1.000	.075	.351
Fibrosis score*	5.2 ± 8.6	3.3 ± 1.4	0.402	4.1 ± 4.0	3.6 ± 2.5	0.426	0.698	0.864
Child-Pugh score (A:B:C)	37:2:0	38:1:0	.840	27:0:2	27:1:1	.514	.124	.492

Data are mean values ± standard deviations or number (%).

*Missing data.

Nominal variables are compared using Fisher's exact tests or Pearson Chi square tests. Continuous variables are compared using paired *t* tests (*P*-value 1 and 2) or Student’s *t* tests (*P*-value 3 and 4). *P*-value 1 and 2 are comparisons before and after 48 weeks of treatment. *P*-value 3 and 4 are comparisons between entecavir and telbivudine groups at baseline and week 48, respectively.

Abbreviations: ALT, alanine aminotransferase; AST, aspartate aminotransferase; HBeAg, hepatitis B virus e antigen; HBV, hepatitis B virus.

Two groups of patients had similar liver fibrosis scores at baseline and at week 48. Intriguingly, 48 weeks of entecavir treatment improved albumin-to-globulin ratio in cirrhotic patients, while telbivudine treatment had an opposite effect. Drastically descends in serum IgA and IgM levels at week 48 were found in both groups of the patients. However, the level of serum IgG declined after 48 weeks of entecavir treatment but remained high in the telbivudine group. A similar result was found when the 6 HBeAg-positive patients in the entecavir group was excluded (S1 Table).

#### Cytokine profiles in cirrhotic patients receiving entecavir or telbivudine treatment

**Fig 1:**
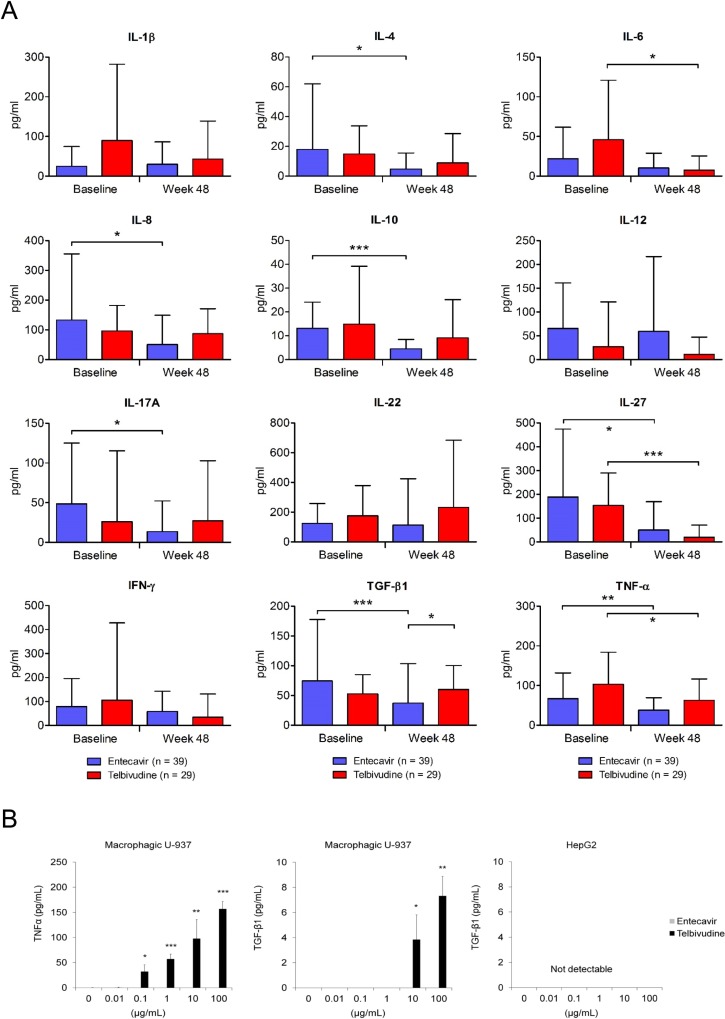
Serum cytokine profiles. (A) Levels of various cytokines in patients with hepatitis B virus-related liver cirrhosis at baseline and after 48 weeks of treatment are shown. The mean value of each group is shown as a black line. Student *t* tests are used for comparing values between entecavir and telbivudine groups at the same time point (lane 1 vs 2; lane 3 vs 4). Paired *t* tests are used for comparing values at baseline and week 48 (lane 1 vs 3; lane 2 vs 4). (B) The level of TGF-β1 or TNFα in macrophagic U-937 or HepG2 cells after 48 hours of telbivudine or entecavir treatment is shown. Data are obtained from three independent experiments and shown as mean with standard deviation. Student *t* tests are used for comparisons between the control group and treatment groups. *P < 0.05, **P < 0.01, ***P < 0.001. IFN, interferon; IL, interleukin; TGF, transforming growth factor; TNF, tumor necrosis factor.

**Fig 2:**
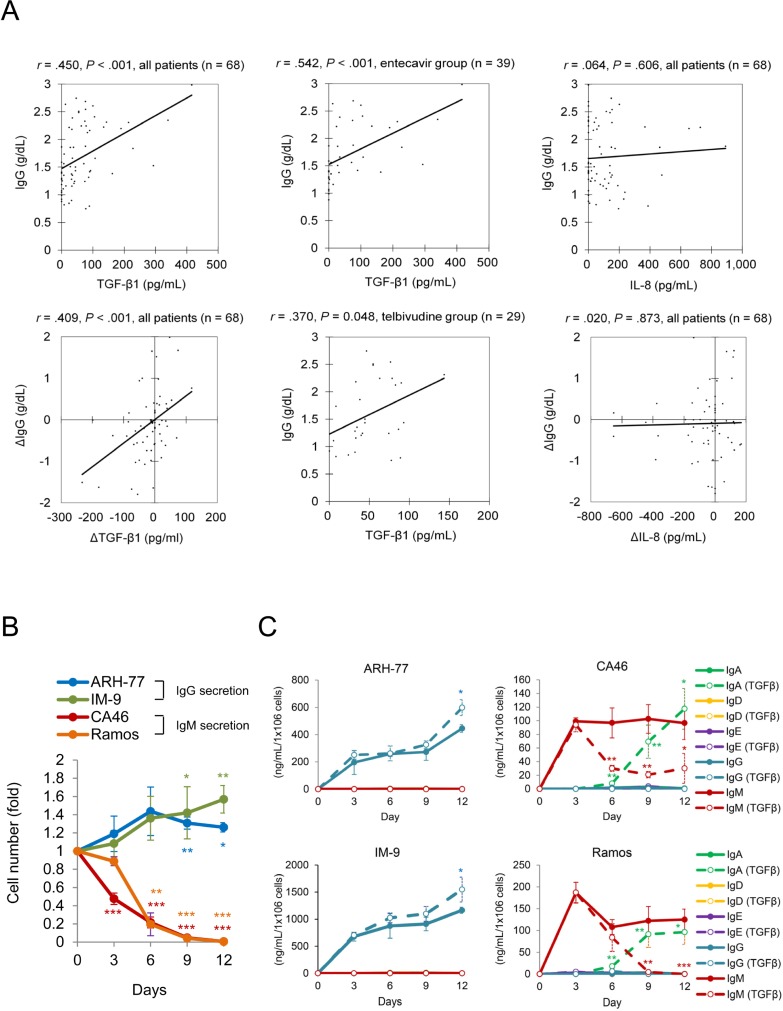
Transforming growth factor (TGF)-β1 induces the proliferation and IgG secretion in IgG-producing cells. (A) Relationships of IgG with TGF-β1 in patients with liver cirrhosis before or during treatment (Δ) are shown as scatter plots and regression lines. The coefficient *r* is taken from Pearson's correlation test. Time course studies for the (B) cell numbers and (C) levels of different immunoglobulin isotypes from different B cell lines after TGF-β1 treatment are shown. Cells were subcultured at a 1:10 dilution and kept in fresh complete media supplemented with 10 ng/mL of TGF-β1 every three days. Concentrations of immunoglobulins are normalized with the cell number. Data are obtained from three independent experiments and shown as mean values with standard deviations. Student *t* tests are used for comparisons (B) between baseline and each time point, and (C) between the control and treatment groups. *P < 0.05, **P < 0.01, ***P < 0.001.

Two groups of patients had approximately equal cytokine profiles at baseline (Fig 1A). Forty-eight weeks of entecavir treatment led to declines in interleukin (IL)-4, IL-8, IL-10, IL-17A, IL-27, transforming growth factor (TGF)-β1, and tumor necrosis factor (TNF)-α levels while telbivudine treatment reduced IL-6, IL-27, and TNFα levels (Fig 1A). At week 48, TGF-β1 level in the telbivudine group (62.9 ± 44.0 pg/mL) was higher than that in the entecavir group (35.0 ± 63.8 pg/mL) (Fig 1A) and 2 drugs had a different potency on the restoration of the expression of IL-1β, IL-6, IL-10, and TGF-β1 (Table 2). Telbivudine stimulated TNFα and TGF-β1 secretion in human macrophagic U-937 cells but not in human hepatoma HepG2 cells (Fig 1B).

**Table 2:** Changes in levels (Δ) of factors after 48 weeks of treatment in patients with HBV-related liver cirrhosis.

Variable	Entecavir (n = 39)	Telbivudine (n = 29)	*P*-value
**Clinical data**			
ALT (U/L)	-21.0 (-484.0–43.0)	-31.0 (-582.0–99.0)	.594
AST (U/L)	-18.0 (-251.0–20.0)	-10.0 (-376.0–89.0)	.336
Albumin (g/dL)	0.2 (-0.9–1.3)	0.1 (-1.1–1.2)	.029
Total globulin (g/dL)	-6.1 (-45.0–27.9)	7.4 (-90.8–51.0)	< .001
Albumin/globulin ratio	0.4 (-1.1–1.4)	-0.2 (-2.1–0.9)	< .001
γ-globulin (g/dL)	-4.9 (-23.1–4.6)	-1.1 (-19.0–17.1)	.003
IgG (g/dL)	-2.0 (-18.0–3.9)	0.0 (-16.4–20.2)	.013
IgA (g/L)	-0.7 (-3.2–2.8)	-0.5 (-2.6–0.6)	.246
IgM (g/L)	-2.2 (-8.0–0.9)	-0.8 (-9.5–0.4)	.018
IgD (μg/dL)	0.0 (-4.0–3.0)	0.0 (-4.0–11.0)	.396
IgE (μg/dL)	0.0 (-3.0–1.0)	0.0 (-2.0–3.0)	.408
Total bilirubin (mg/dL)	-0.1 (-5.6–0.8)	-0.1 (-1.9–0.5)	.693
HBV DNA (Log_10_ IU/mL)	-5.0 (-8.0–1.7)	-5.8 (-8.0–0.2)	.135
**Cytokine (pg/mL)**			
IL-1β	0.0 (-210.2–180.0)	-11.3 (-1033.5–431.2)	.032
IL-4	0.0 (-192.7–8.6)	0.0 (-54.2–78.4)	.814
IL-6	-2.3 (-190.4–35.7)	-18.5 (-334.6–45.7)	.014
IL-8	-14.0 (-654.0–169.0)	0.0 (-171.2–141.0)	.225
IL-10	-6.0 (-45.7–7.3)	0.0 (-110.6–63.0)	.018
IL-12p70	-9.3 (-365.3–910.7)	0.0 (-457.3–173.3)	.076
IL-17A	0.0 (-238.0–176.7)	0.0 (-322.3–351.0)	.089
IL-22	-49.0 (-410.2–1388.4)	-39.5 (-176.7–1074.4)	.292
IL-27	0.0 (-939.4–511.7)	-136.3 (-337.5–0.0)	.059
IFN-γ	0.0 (-573.8–212.5)	0.0 (-1577.2–275.0)	.594
TGF-β1	-14.0 (-232.8–34.0)	11.3 (-61.9–118.0)	< .001
TNF-α	-11.8 (-202.0–51.0)	-17.8 (-186.8–122.6)	.413

All data are shown as median (range).

*P*-values are obtained from Mann-Whitney *U* tests.

Abbreviations: ALT, alanine aminotransferase; AST, aspartate aminotransferase; HBV, hepatitis B virus; IFN, interferon; IL, interleukin; TGF, transforming growth factor; TNF, tumor necrosis factor.

#### TGF-β1 induces cell proliferation and IgG overproduction

Next, we assessed if any cytokine was associated with telbivudine-related IgG overproduction. Serum IgG level in cirrhotic patients either at baseline or during the treatment was associated with the level of TGF-β1 (Fig 2A). Multivariate logistic regression analyses revealed that TGF-β1 was an independent factor that was associated with IgG hyperactivity at baseline or post-treatment increment (Table 3). Results from time course assays showed that recombinant human TGF-β1 treatment promoted cell proliferation and IgG secretion in IgG-producing IM-9 and ARH-77 cells (Fig 2B and 2C). However, TGF-β1 treatment perished IgM-producing CA46 and Ramos cells, reduced IgM secretion, and facilitated Ig isotype switch from IgM to IgA. Together with clinical observations and *in vitro* assays, telbivudine treatment upregulates TGF-β1, which stimulates IgG overproduction and may lead to IgG-related hypergammaglobulinemia.

**Table 3:** Multivariate logistic regression analysis of factors that are associated with the hyperactivity (> 2 g/dL) or post-treatment increment of IgG in patients with HBV-related liver cirrhosis (n = 68).

	IgG hyperactivity			IgG increment after treatment	
Variable (Baseline level)	Odds ratio (95% CI)	*P*-value	Variable (Changes in levels, Δ)	Odds ratio (95% CI)	*P*-value
**Clinical data**			**ΔClinical data**		
Sex (Male 1, female 0)	1.617 (0.090–29.018)	.744			
Age (years)	0.920 (0.806–1.049)	.212			
ALT (U/L)	0.957 (0.914–1.002)	.061	ALT (U/L)	1.008 (0.989–1.026)	.416
AST (U/L)	1.075 (0.997–1.160)	.060	AST (U/L)	0.980 (0.951–1.011)	.200
Albumin (g/dL)	4.998 (0.482–51.870)	.178	Albumin (g/dL)	0.718 (0.101–5.098)	.741
Globulins (g/dL)	1.102 (0.993–1.223)	.068	Globulins (g/dL)	1.005 (0.979–1.032)	.702
Total bilirubin (mg/dL)	1.926 (0.660–5.618)	.230	Total bilirubin (mg/dL)	2.145 (0.758–6.070)	.150
HBV DNA (Log_10_ IU/mL)	2.726 (0.876–8.482)	.083	HBV DNA (Log_10_ IU/mL)	1.126 (0.764–1.660)	.548
**Cytokine (pg/mL)**			**ΔCytokine (pg/mL)**		
IL-1β	1.023 (1.004–1.043)	.017	IL-1β	1.002 (0.995–1.009)	.631
IL-4	0.990 (0.962–1.019)	.514	IL-4	0.990 (0.967–1.013)	.371
IL-6	0.943 (0.894–0.995)	.033	IL-6	1.005 (0.991–1.020)	.460
IL-8	0.991 (0.984–0.999)	.034	IL-8	0.997 (0.992–1.002)	.244
IL-10	0.969 (0.917–1.023)	.248	IL-10	0.994 (0.962–1.027)	.716
IL-12p70	0.999 (0.983–1.015)	.881	IL-12p70	0.998 (0.993–1.003)	.472
IL-17A	1.003 (0.990–1.016)	.663	IL-17A	1.002 (0.994–1.009)	.682
IL-22	1.006 (0.998–1.015)	.149	IL-22	1.000 (0.998–1.003)	.727
IL-27	1.006 (0.999–1.013)	.074	IL-27	1.001 (0.999–1.004)	.377
IFN-γ	0.962 (0.915–1.011)	.123	IFN-γ	1.000 (0.997–1.003)	.891
TGF-β1	1.054 (1.014–1.097)	.008	TGF-β1	1.023 (1.003–1.044)	.025
TNF-α	0.996 (0.979–1.013)	.632	TNF-α	1.003 (0.991–1.014)	.652

Abbreviations: Δ, changes in levels from baseline to week 48 after treatment. ALT, alanine aminotransferase; AST; aspartate aminotransferase; CI, confidence interval; HBV, hepatitis B virus; IFN, interferon; IL, interleukin; TGF, transforming growth factor; TNF, tumor necrosis factor.

#### IgG-Fc agalactosylation is associated with TGF-β1 and IgG overproduction

**Fig 3:**
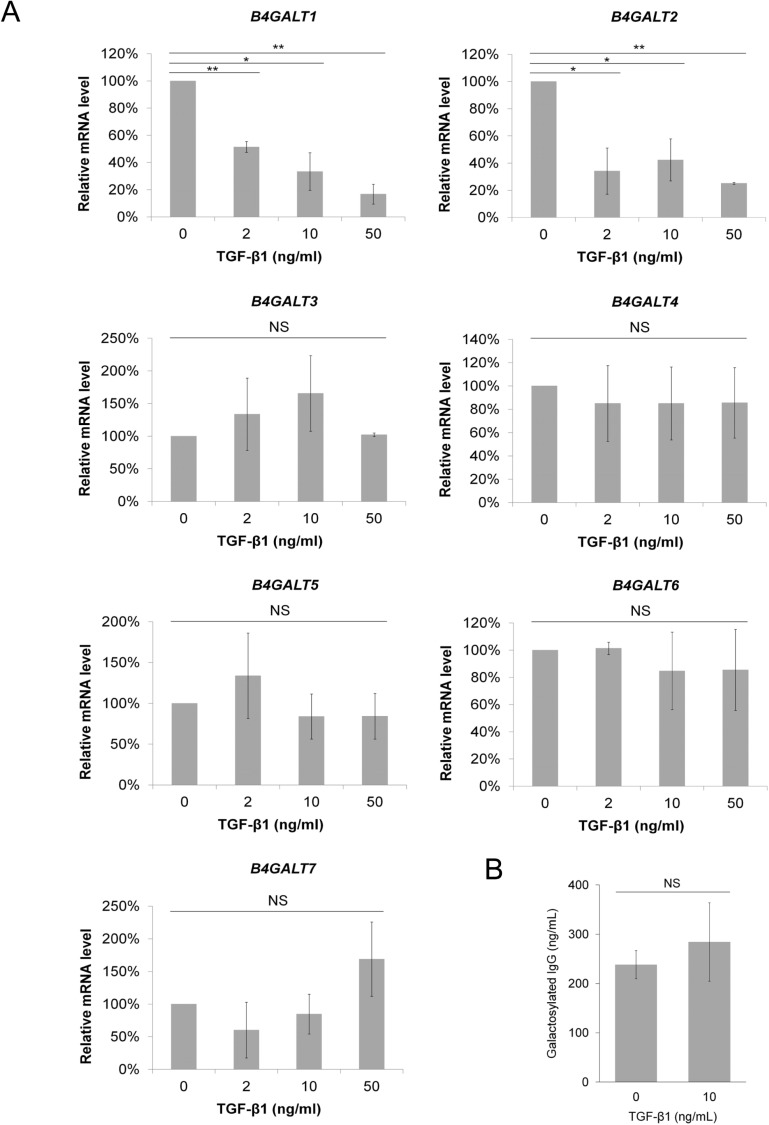
Association of transforming growth factor (TGF)-β1 and IgG agalactosylation. (A) Messenger RNA levels of β-1,4-galactosyltransferases (b4galts) after TGF-β1 treatment for 24 hours, and (B) the concentration of agalactosylated IgG (the percentage of total agalactosylated glycoforms × cell number) that was secreted from IM-9 cells after 10 ng/mL of TGF-β1 treatment for 12 days are shown. Data are obtained from three independent experiments and shown as mean values with standard deviations. Student *t* tests are used for comparisons between the control and treatment groups. *P < 0.05, **P < 0.01, NS, not significant.

An increase in the proportion of serum agalactosylated IgG is a typical seromarker for liver cirrhosis. Results from *N*-glycan analyses showed that 2 groups of the patients had a similar IgG glycan pattern at baseline (Table 4). After 48 weeks of treatment, the telbivudine group had higher proportions of G0F (agalactosylated and fucosylated) and G0FN (G0F with a bisecting *N*-acetylglucosamine) glycoforms but lower proportions of G2FS (fully galactosylated, fucosylated, and sialylated) and G2FNS (G2FS with a bisecting *N*-acetylglucosamine) glycoforms on IgG than the entecavir group (Table 4). In general, telbivudine treatment had an opposite effect on IgG agalactosylation (G0F + G0 + G0FN) to entecavir treatment in cirrhotic patients. The trend of agalactosylated IgG during treatment was correlated with that of TGF-β1 (*r* = 0.291, *P* = 0.016) and IgG concentrations (*r* = 0.380, *P* = 0.001) (Table 5). Based on these observations, we examined whether TGF-β1 regulates β-1,4-galactosyltransferases (*B4GALT*s), a gene family that contributes to galactose editing, in IM-9 cells and found that TGF-β1 treatment downregulated messenger RNA levels *B4GALT1 and B4GALT2* but not other 5 *B4GALT*s (Fig 3A). Intriguingly, TGF-β1 showed no sign of changing an overall concentration of galactosylated IgG that was synthesized from IM-9 cells (Fig 3B). These findings, together with the effect of TGF-β1 on IgG overproduction, indicate that the galactose editing in B cells remained constant upon TGF-β1 treatment and TGF-β1-related increase in the proportion of agalactosylated IgG was caused by an enhanced total IgG level.

**Table 4:** Serum IgG_1_-Fc *N*-glycan profiles in patients with hepatitis B virus-related liver cirrhosis.

Glycan composition (deduced glycoform)	Entecavir group (n = 39)			Telbivudine group (n = 29)				
	Baseline	Week 48	*P*-value^1^	Baseline	Week 48	*P*-value^2^	*P*-value^3^	*P*-value^4^
Hex3HexNAc4dHex1 (G0F)	35.9 ± 8.5	31.5 ± 7.3	< .001	32.5 ± 7.2	35.3 ± 7.9	.032	.090	.044
Hex3HexNAc4 (G0)	2.1 ± 1.9	2.0 ± 1.9	.627	2.5 ± 1.1	2.5 ± 1.0	.686	.284	.275
Hex3HexNAc5dHex1 (G0FN)	3.4 ± 1.6	2.9 ± 1.6	.146	4.3 ± 2.1	4.9 ± 2.1	.007	.051	< .001
Hex4HexNAc4dHex1 (G1F)	31.7 ± 3.9	31.9 ± 5.7	.906	32.3 ± 3.6	31.3 ± 3.5	.136	.582	.611
Hex4HexNAc5dHex1 (G1FN)	5.8 ± 2.6	6.3 ± 5.8	.567	5.2 ± 1.7	5.3 ± 1.6	.649	.287	.397
Hex4HexNAc4dHex1NeuAc1 (G1FS)	0.8 ± 0.8	1.2 ± 1.2	.141	0.9 ± 0.3	1.0 ± 0.4	.907	.393	.299
Hex5HexNAc4dHex1 (G2F)	11.4 ± 3.4	12.1 ± 4.2	.132	13.8 ± 4.3	11.9 ± 3.7	.001	.010	.859
Hex5HexNAc4dHex1NeuAc1 (G2FS)	8.2 ± 3.1	11.2 ± 8.3	.035	7.2 ± 2.4	6.7 ± 3.1	.278	.145	.003
Hex5HexNAc5dHex1 (G2FN)	0.5 ± 0.6	0.9 ± 1.5	.202	1.0 ± 0.6	1.0 ± 1.2	.856	.002	.761
Hex5HexNAc5dHex1NeuAc1 (G2FNS)	0.1 ± 0.4	0.1 ± 0.1	.240	0.2 ± 0.4	0.1 ± 0.1	.167	.637	.048
Total agalactosylation (G0F + G0 + G0FN)	41.4 ± 9.4	36.5 ± 8.6	< 0.001	39.4 ± 8.2	42.8 ± 9.5	.018	.357	.006

Data are shown in mean values ± standard deviations.

Variables are compared using paired *t* tests (*P*-value 1 and 2) or Student’s *t* tests (*P*-value 3 and 4). *P*-value 1 and 2 are comparisons before and after 48 weeks of treatment. *P*-value 3 and 4 are comparisons between entecavir and telbivudine groups at baseline and week 48, respectively.

Abbreviations: dHex or F, fucose; G0, agalactosylation; G1, partial galactosylation; G2, full galactosylation; Hex, hexose; NAc or N, *N*-acetylglucosamine; NeuAc or S, sialic acid.

**Table 5:** Correlations of changes in levels of serum IgG_1_-Fc *N*-glycoforms and TGF-β1 or IgG in cirrhotic patients (n = 68) during treatment.

Glycan composition	TGF-β1		IgG	
(deduced glycoform)	Coefficient r	*P*-value	Coefficient r	*P*-value
Hex3HexNAc4dHex1 (G0F)	.245	.044	.335	.005
Hex3HexNAc4 (G0)	.170	.166	-.049	.690
Hex3HexNAc5dHex1 (G0FN)	.179	.144	.324	.007
Hex4HexNAc4dHex1 (G1F)	-.026	.836	-.267	.028
Hex4HexNAc5dHex1 (G1FN)	.093	.449	.013	.915
Hex4HexNAc4dHex1NeuAc1 (G1FS)	-.206	.093	.032	.794
Hex5HexNAc4dHex1 (G2F)	-.252	.038	-.391	< .001
Hex5HexNAc4dHex1NeuAc1 (G2FS)	-.261	.032	-.069	.576
Hex5HexNAc5dHex1 (G2FN)	.183	.135	-.018	.882
Hex5HexNAc5dHex1NeuAc1 (G2FNS)	.027	.830	.077	.533
Total agalactosylation (G0F + G0 + G0FN)	.291	.016	.380	.001

Abbreviations: dHex or F, fucose; G0, agalactosylation; G1, partial galactosylation; G2, full galactosylation; Hex, hexose; NAc or N, *N*-acetylglucosamine; NeuAc or S, sialic acid; TGF, transforming growth factor.

## Discussion

In this study, 48 weeks of telbivudine therapy rather than entecavir therapy was found to be associated with TGF-β1 hyperactivity, IgG-related hypergammaglobulinemia, and a decrease in the proportion of serum IgG-Fc galactosylation in the patients with HBV-related liver cirrhosis. Clinical reports disclose that telbivudine has a compatible efficacy on HBV suppression and even a higher efficacy on HBV e antigen seroconversion than entecavir for chronic HBV infection [11–14]. Moreover, telbivudine treatment induces TNF-α, IL-4, IL-12, and interferon-γ expressions in a murine hepatitis virus type 3-infected macrophage model [15, 16]. Our results showed that telbivudine treatment stimulated TGF-β1secretion in human macrophagic U-937 cells, which reflects the clinical observation from the cirrhotic patients. These clues imply that telbivudine possesses an immunomodulatory activity. A slight increase in TGF-β1 after telbivudine treatment is the net result that comes from a delicate balance between pharmacology and immunomodulation. It is very likely that other cell lineages or complex regulatory networks may involve in telbivudine-dependent TGF-β1 upregulation *in vivo*. TGF-β1 is a notoriously liver fibrogenic factor that activates quiescent hepatic stellate cells to produce and accumulate extracellular matrix proteins in the liver tissues [17–19]. Apart from TGF-β1, an accumulation of immune complexes by a plethora of IgG antibodies tends to induce hepatic fibrosis as well [20, 21]. Accordingly, it is rational to speculate that TGF-β1 and IgG form a vicious circle for the progression of liver fibrosis. From a clinical aspect, long-term follow-ups for TGF-β1 kinetics in addition to HBV virology and liver histology would be of great importance to predict the status of liver stiffness and related complications in cirrhotic patients after years.

Hypergammaglobulinemia is an immunoproliferative disease and frequently linked to chronic granulomatous inflammations, multiple myelomas, lymphomas, infection diseases, autoimmune disorders, and liver cirrhosis [22, 23]. Patients with hypergammaglobulinemia have a weak immunity against infections, an enlarged lymphatic system, hepatosplenomegaly, or tissue damages by the deposition of immune complexes. Several theories regarding cirrhosis-related hypergammaglobulinemia have been postulated. First, antibodies after immunization with microorganisms are vigorously synthesized due to the influx of gut bacteria by portal hypertension and weak filtration of the cirrhotic liver [24, 25]. Second, inefficient turnover and removal of immunoglobulins by the malfunctioned liver cause antibody accumulation [23, 26]. Third, B cell clones, for some reasons, generally activate to secrete antibodies in an antigen-independent mode [23]. Excessive IgM production is the most common cause of hypergammaglobulinemia in a hereditary condition or acute infection. Nevertheless, various cohort reports refer the virus infection or cirrhosis-related hypergammaglobulinemia to IgG overproduction [22, 27, 28]. Our findings demonstrated that TGF-β1, in addition to inhibit IgM secretion and stimulate antibody isotype switching to IgA [29–32], also enhances IgG production. Of course, peripheral B cell is an ideal model for investigating the underpinning of TGF-β1-mediated IgG overproduction. However, priming stimuli for primary CD19^+^ B cell activation, for example, IL-10 or CD40 ligand, are known to interfere antibody class switching and may strikingly conceal the effect of ensuing treatment [33]. Therefore, we chose B cell lines that continually secrete IgM or IgG as a model in the present study. Our results mentioning the impact of TGF-β1 on IgM secretion were coincident with previous reports. Additionally, TGF-β1 augmented the proliferation and IgG secretion from IgG-producing ARH-77 and IM-9 cells, indicating that TGF-β1 promotes IgG secretion in a direct manner. Assuredly, we know that *in vitro* studies may not totally reflect the divergence of B cell clones and interplay between the hepatic microenvironment and circulation during liver cirrhosis.

The *N*-glycosylation pattern is phylogenetically heterogeneous in IgG. Several lines of evidence [34, 35] and our previous report [7] point out an increase in the proportion of serum agalactosylated IgG during liver cirrhosis. Herein we demonstrated that the percentage of serum IgG agalactosylation was correlated with the level of TGF-β1. However, the redundancy of b4galts maintains the yield of galactosylated IgG in the presence of TGF-β1. The influence of TGF-β1 on IgG agalactosylation may build upon a dilution in the proportion of galactosylated IgG as a consequence of a crude glycosylation in B lymphocytes when producing a huge amount of IgG proteins. This hypothesis could be supported by other reports of which enzymatic activities and protein levels of galactosyltransferases in B lymphocytes in patients with rheumatoid arthritis and healthy individuals were identical [36–38].

Pathologywise, liver cirrhosis is not a single disease entity and patients often suffer from impaired hepatic functions and also a broad range of complications that injure multiple organs. Unfortunately, so far effective therapy for curing severe fibrosis, distorted vascular architecture, or hepatic malfunction has not been developed yet. Putting things in perspective, a combined remedy with antiviral agents plus TGF-β1 inhibitor is postulated to afford a better efficacy on the alleviation of liver stiffness and related immunopathies for patients with HBV-related liver cirrhosis.

## Supporting information

S1 Table (DOCX)

## Abbreviations

ALT: alanine aminotransferase
AST: aspartate aminotransferase
b4galt: β-1,4-galactosyltransferase
CD: cluster of differentiation
CI: confidence interval
ELISA: enzyme-linked immunosorbent assay
F for glycan: fucosylation
Fc: crystallizable fragment
G0 for glycan: agalactosylated
G1 for glycan: partially galactosylated
G2 for glycan: fully galactosylated
N or GlcNAc: *N*-acetylglucosamine
HBeAg: hepatitis B virus e antigen
HBV: hepatitis B virus
IFN: interferon
Ig: immunoglobulin
IL: interleukin
LC-MS/MS: liquid chromatography–tandem mass spectrometry
qRT-PCR: quantitative reverse transcription-polymerase chain reaction
TGF: transforming growth factor
TNF: tumor necrosis factor
S for glycan: sialylated
ULN: upper limit of normal.
