# Supplementary material for: Telbivudine on IgG-associated hypergammaglobulinemia and TGF-β1 hyperactivity in hepatitis B virus-related liver cirrhosis
Source: PLoS One. 2019 Nov 26;14(11):e0225482. doi: 10.1371/journal.pone.0225482 (PMC6879168; doi:10.1371/journal.pone.0225482)
Supplement: S1 Table — (DOCX) [file pone.0225482.s001.docx]

**Supplementary Table 1.** Clinical data of HBeAg (-) patients with HBV-related liver cirrhosis.

|  | Entecavir group (n = 33) | | |  | Telbivudine group (n = 29) | | |  |  |
| --- | --- | --- | --- | --- | --- | --- | --- | --- | --- |
| Variable | Baseline | Week 48 | *P*-value^1^ |  | Baseline | Week 48 | *P*-value^2^ | *P*-value^3^ | *P*-value^4^ |
| Male, no. (%) | 19 (57.6) |  |  |  | 22 (75.9) |  |  | .180 |  |
| Age (years) | 54.8 ± 8.9 |  |  |  | 56.7 ± 12.0 |  |  | .469 |  |
| ALT (U/L) | 73.3 ± 89.2 | 31.9 ± 14.6 | .013 |  | 116.7 ± 135.2 | 40.4 ± 30.6 | .005 | .137 | .158 |
| AST (U/L) | 62.0 ± 47.3 | 33.2 ± 10.3 | .001 |  | 89.5 ± 91.9 | 45.1 ± 27.2 | .013 | .155 | .024 |
| Albumin(g/dL) | 4.0 ± 0.5 | 4.2 ± 0.4 | < .001 |  | 4.0 ± 0.6 | 4.1 ± 0.5 | .487 | .550 | .440 |
| Total globulin (g/dL) | 3.8 ± 1.5 | 3.2 ± 1.0 | .051 |  | 4.1 ± 2.8 | 4.8 ± 1.5 | .273 | .546 | < .001 |
| Albumin/Globulin ratio | 1.2 ± 0.4 | 1.4 ± 0.4 | .010 |  | 1.3 ± 0.6 | 0.9 ± 0.3 | .014 | .491 | < .001 |
| γ-globulin (g/dL) | 2.2 ± 0.6 | 1.5 ± 0.5 | < .001 |  | 1.9 ± 0.6 | 1.9 ± 0.6 | .977 | .113 | .002 |
| IgG (g/dL) | 1.7 ± 0.5 | 1.4 ± 0.5 | .001 |  | 1.6 ± 0.6 | 1.8 ± 0.6 | .188 | .352 | < .001 |
| IgA (g/L) | 1.3 ± 1.0 | 0.4 ± 0.9 | < .001 |  | 0.9 ± 0.8 | 0.2 ± 0.2 | < .001 | .105 | .272 |
| IgM (g/L) | 3.1 ± 1.5 | 0.8 ± 0.6 | < .001 |  | 2.5 ± 2.5 | 0.8 ± 0.4 | < .001 | .248 | .763 |
| IgD (μg/L) | 2.7 ± 2.3 | 2.4 ± 1.8 | .109 |  | 2.8 ± 2.8 | 3.1 ± 3.0 | .577 | .873 | .283 |
| IgE (μg/L) | 2.7 ± 1.2 | 2.3 ± 0.7 | .005 |  | 2.7 ± 1.2 | 2.6 ± 1.4 | .595 | .930 | .188 |
| Total bilirubin (mg/dL) | 1.2 ± 0.6 | 1.1 ± 0.4 | .203 |  | 1.3 ± 0.7 | 1.2 ± 0.5 | .144 | .474 | .241 |
| HBV DNA (Log_10_ IU/mL) | 5.4 ± 1.4 | 1.0 ± 1.7 | < .001 |  | 5.9 ± 1.2 | 0.4 ± 1.3 | < .001 | .171 | .134 |
| Virological response |  | 24 (72.7) |  |  |  | 25 (86.2) |  |  | .193 |
| Child-Pugh score (A:B:C) | 28:1:0 | 28:1:0 | 1.000 |  | 27:0:2 | 27:1:1 | .514 | .221 | .601 |

Data are mean values ± standard deviations or number (%).

Nominal variables are compared using Fisher's exact tests or Pearson Chi square tests. Continuous variables are compared using paired *t* tests (*P*-value 1 and 2) or Student’s *t* tests (*P*-value 3 and 4). *P*-value 1 and 2 are comparisons before and after 48 weeks of treatment. *P*-value 3 and 4 are comparisons between entecavir and telbivudine groups at baseline and week 48, respectively.

Abbreviations: ALT, alanine aminotransferase; AST, aspartate aminotransferase; HBeAg, hepatitis B virus e antigen; HBV, hepatitis B virus.
